# From bedside to bench: the needs of clinically-relevant animal models of disease development

**DOI:** 10.1186/1878-5085-5-S1-A96

**Published:** 2014-02-11

**Authors:** R Andrew Tasker

**Affiliations:** 1 Department of Biomedical Sciences, University of Prince Edward Island, Charlottetown, PEI, Canada

## 

Identification of new drug targets and the development of new drugs and therapies for neurological diseases currently relies on preclinical data generated using *in vitro* and *in vivo* animal models as a prelude to clinical trials. While this “bench to bedside” approach has resulted in some successes, it has been notoriously inefficient, largely due to the failure of most preclinical results to translate to clinical use. Countless new chemical entities have shown promise in preclinical studies but have then either failed to demonstrate efficacy in Phase 2 or Phase 3 clinical trials or have been withdrawn due to unanticipated side effects or toxicities. These failures not only limit the availability of new therapeutic options but they can be catastrophically expensive thereby curtailing future research and driving up the cost of developing new agents. An oft-cited example is neuroprotection following stroke, wherein literally thousands of new compounds have shown robust neuroprotection in animal studies, but to date, none have translated to a clinically useful compound despite billions of euros spent.

While it is clear that part of the problem with current animal models is that the brain of each species is different (rats and mice are not humans and vice versa) it is also my contention that there are fundamental problems with our historical approach to developing and utilizing animal models for new drug development. These problems include:

1. Current animal models reproduce end-stage symptoms instead of disease. This means that it is only possible to test products that mitigate the symptoms that appear late in the disease process. Creation of animal models of disease versus symptoms will permit the identification of early-stage biomarkers and the development of disease-modifying therapies that can be used presymptomatically to slow or arrest the disease process.

2. Models are simple whereas diseases are complex. Most preclinical approaches are essentially “reductionist”; looking at only a single brain region or even cell type. Moreover they are “neurocentric” avoiding the contribution of other cell types (e.g. glia) and other systems (e.g. vasculature). They also tend to be “static” whereas disease progression is “dynamic” and subject to on-going modification.

3. Most models concentrate on one set of symptoms associated with one defined disease. In contrast, most neurology patients present with a complex milieu of symptoms and co-morbidities that combine features of multiple diseases. Further, each patient’s presentation is often different, so to progress therapy must move beyond the “one size fits all” mentality and accordingly animal models must embrace diversity rather than trying to eliminate it.

This presentation will expand upon these somewhat provocative ideas and will introduce several examples of new animal models that are trying to overcome these problems. Such models are essential if we are to develop new methodologies and screening techniques that are predictive of disease onset, are aimed at prevention rather than palliation, and recognize the patient as a person. Equally important is the need for improved dialogue between clinicians and basic scientists so that future preclinical drug development is based on what disease looks like in the patient rather than the test-tube.

